# VISTA: an immune regulatory protein checking tumor and immune cells in cancer immunotherapy

**DOI:** 10.1186/s13045-020-00917-y

**Published:** 2020-06-29

**Authors:** Xing Huang, Xiaozhen Zhang, Enliang Li, Gang Zhang, Xun Wang, Tianyu Tang, Xueli Bai, Tingbo Liang

**Affiliations:** 1 Zhejiang Provincial Key Laboratory of Pancreatic Disease, the First Affiliated Hospital, School of Medicine, Zhejiang University, 79 Qingchun Road, Hangzhou, 310003 Zhejiang China; 2 Department of Hepatobiliary and Pancreatic Surgery, the First Affiliated Hospital, School of Medicine, Zhejiang University, Hangzhou, 310003 Zhejiang China; 3 Innovation Center for the Study of Pancreatic Diseases of Zhejiang Province, Hangzhou, 310003 Zhejiang China

**Keywords:** Cancer immunotherapy, Co-inhibition, Co-stimulation, Immune checkpoint, VISTA

## Abstract

VISTA (V-domain immunoglobulin suppressor of T cell activation) is a well-established immune regulatory receptor. However, pre-clinical investigations indicated more complicated influences of VISTA on cancer immunity than previously recognized. Here, we review the current knowledge on the therapeutic phenotypes and molecular mechanisms that underlie the contradictory roles of VISTA in checking anti-cancer immune responses. Furthermore, we highlight the potential indeterminacy of VISTA-targeted strategies in cancer immunotherapy, with in silico analyses. In fact, VISTA functions like a homeostatic regulator that actively normalizes immune responses. Thus, the regulatory role of VISTA in anti-cancer immunity remains to be fully elucidated.

## Background

Immunotherapies, including but not limited to, passive immunization using donor T cells, immunoadjuvants or cytokines with immunomodulatory properties, vaccines, chimeric antigen receptor T cell (CAR-T), and immune checkpoint blocking antibodies, have been regarded as some of the most effective strategies in the treatment of multiple human cancers in the past few decades [1, 2]. Among these strategies, immune checkpoint blockade is becoming a cutting edge approach to cancer immunotherapy [3]. Numerous studies have been performed to elucidate the mechanism and therapeutic potential of representative immune checkpoints, such as cytotoxic T lymphocyte-associated antigen-4 (CTLA-4), and programmed death receptors, such as programmed cell death protein 1 (PD-1). In addition, the discovery of novel targets with immune checkpoint activity has provided new opportunities for the systematic investigation of cancer immunoregulatory networks and also represents a potential breakthrough in the development of promising therapeutic drugs.

V-domain immunoglobulin suppressor of T cell activation (VISTA, also known as c10orf54, VSIR, SISP1, B7-H5, PD-1H, DD1α, Gi24, and Dies1) has become a current focus of research [4]. VISTA is primarily expressed in hematopoietic cells. For example, in leukocytes, the highest levels of VISTA protein expression are found in myeloid cells, particularly microglia and neutrophils followed by monocytes, macrophages, and dendritic cells [5]. Within the T lymphocyte compartment, VISTA is most highly expressed on naïve CD4+ and Foxp3+ regulatory T cells [6]. Moreover, its expression on cancer cells has also been evaluated (described in detail below).

**Fig. 1:**
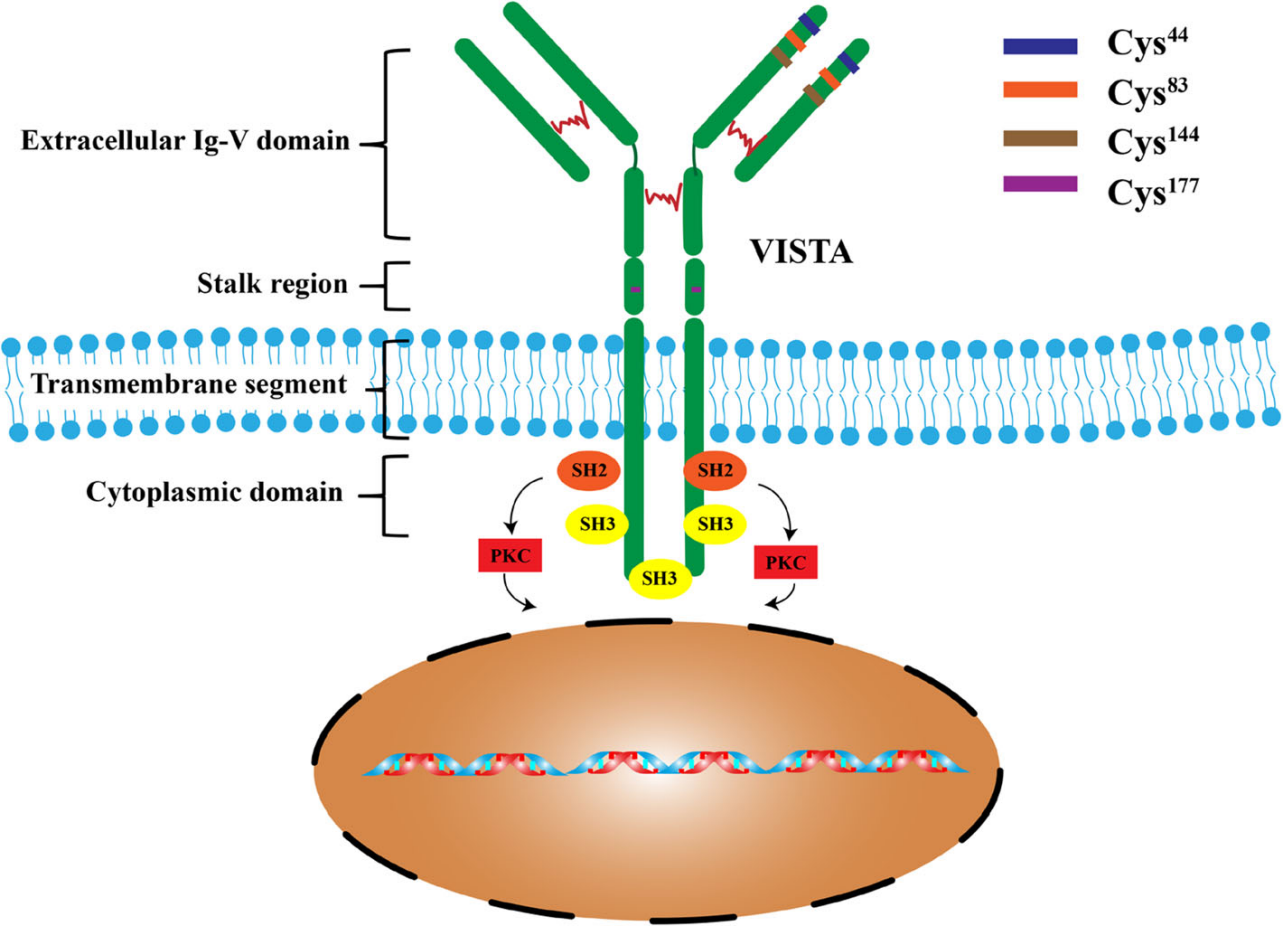
VISTA structure and its downstream signaling

VISTA is a type I transmembrane protein consisting of a single N-terminal immunoglobulin (Ig) V-domain, a stalk of approximately 30 amino acids (aa), a transmembrane domain, and a 95-aa cytoplasmic tail [7]. Analysis of the IgV domain of VISTA shows that this region has the greatest homology with programmed death ligand 1 (PD-L1). The IgV domain of VISTA possesses a canonical disulfide bond between the putative B and F strands. However, uniquely, it has four additional invariant cysteines [4]. Within the conserved cytoplasmic tail, VISTA resembles CD28 and CTLA-4 but does not possess a classic ITIM/ITAM motif, distinguishing it from other B7 co-receptor molecules. VISTA has a conserved Src homology 2 (SH2)-binding (YxxQ, potentially capable of binding STAT proteins) motif in the middle of the cytoplasmic tail and three C-terminal SH3-binding domains (PxxP, two in CD28 and one in CTLA-4). Although VISTA lacks recognized ITIM or ITSM motifs in the cytoplasmic domain, the protein sequence contains potential protein kinase C binding sites and a proline-rich motif, which may function as a platform to interact with other complexes (Fig. 1). The notion that VISTA functions as a ligand is also based on the observation that a VISTA-Ig fusion protein inhibited anti-CD-3 stimulated proliferation of mouse and human CD4 and CD8 T cells as well as the production of IFNγ and IL-2 [4, 8]. Thus, VISTA can act as both a ligand and receptor in regulating immune responses [7–12].

VISTA was identified as a V-set receptor that suppresses T cell-associated response for immune evasion and survival in several human cancers, such as prostate cancer, non-small cell lung cancer (NSCLC), and colorectal carcinoma [13–15]. However, there is also compelling evidence indicating that VISTA has more complicated influences on cancer immunity than was previously recognized, which does not support the use of VISTA as a target for immunotherapy. In fact, in several specific cancer types, VISTA also plays stimulatory checkpoint-like roles in the activation of anti-cancer immune responses. Thus, in this review, we have summarized the current literature describing VISTA-targeted cancer immunotherapy, highlighting the significance of further precise evaluations of the feasibility of VISTA-based therapeutic strategies based on in silico analyses.

## VISTA as an inhibitory immune checkpoint

**Fig. 2:**
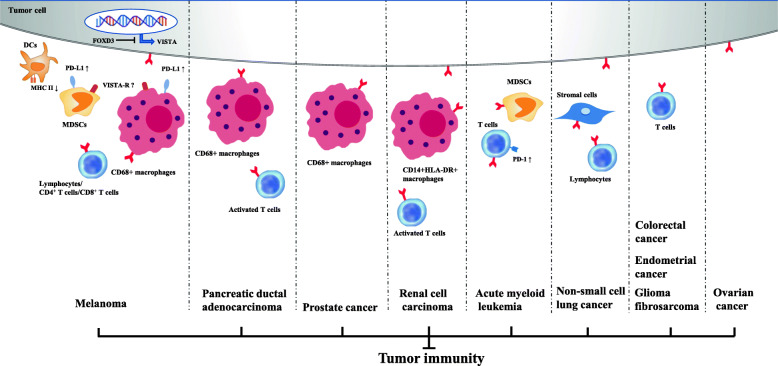
Inhibitory immune checkpoint roles of VISTA in anti-cancer immunity. Positive expression of VISTA on tumor cells and/or immune cells induces an immunosuppressive environment in multiple cancer types

As mentioned above, the function of VISTA in immune regulation is complex and controversial. VISTA not only acts as a ligand expressed on antigen-presenting cells, but also functions as a receptor on T cells. To date, most studies have described the suppressive effect of VISTA on the immune system and the ability of VISTA-deficiency or anti-VISTA treatment to upregulate immune responses [16] (Fig. 2 and Table 1).

**Table 1:** Inhibitory immune checkpoint roles of VISTA

Cancer type	Research object	VISTA expression	Reference
Melanoma	Samples from patients with untreated metastatic melanoma	CD68+ macrophages	Blando et al. [17]
	B16-BL6 melanoma cells	Tumor-associated myeloid cells	Xu et al. [18]
	Patient samples, melanoma cell lines	Melanoma cells	Rosenbaum et al. [19]
	B16 OVA melanoma models	CD8+ T cells	Kondo et al. [20]
	VISTA-KO mice, PD-1 KO mice, VISTA/PD-1 double KO mice	T cells	Liu et al. [21]
	Patient samples with acquired anti-PD-1 resistance	Lymphocytes	Kakavand et al. [22]
Pancreatic ductal adenocarcinoma	Patient samples	CD68+ macrophages	Blando et al. [17]
	Patient samples	Activated T cells	Xie et al. [23]
	Pancreatic tissue including pancreatic adenocarcinomas	Normal ductal epithelium within the pancreas	Byers et al. [24]
Prostate cancer	Samples from patients with or without ipilimumab treatment	Independent subsets of macrophages	Gao et al. [14]
Renal cell carcinoma	Patient samples	Activated T cells	Ni et al. [25]
	Patient samples	Tumor tissues, CD14+HLA-DR+ macrophages	Hong et al. [26]
Non-small cell lung cancer	NSCLC FFPE tumor samples	NSCLC tumor and stromal cells	Villarroel-Espindola et al. [13]
	NSCLC FFPE tumor samples	NSCLC tumor and stromal cells	Hernandez-Martinez et al. [27]
	Resected tissues and bronchoalveolar lavage samples	Lymphocytes	Brcic et al. [28]
Acute myeloid leukemia	Human AML donors, AML mouse model, VISTA-KO mice	Myeloid subsets and T cells	Kim et al. [29]
	Peripheral blood from AML patients	Myeloid-derived suppressor cells	Wang et al. [30]
Colorectal cancer	VISTA-KO mice, CT26 colon carcinoma cell line	Tumor-infiltrating leukocytes	Xie et al. [15]
Ovarian cancer	Patient samples, ID8 mouse ovarian cancer cell line, mice	Tumor cells	Mulati et al. [31]
Endometrial cancer	Patient samples, OV2944-HM-1 mouse ovarian cancer cell line, mice	Tumor cells, CD8+ T cells	Mulati et al. [31]
Fibrosarcoma	MCA105 fibrosarcoma cell lines, mice	Hematopoietic cell types	Wang et al. [4]
Glioma	Murine glioma model, VISTA-KO mice	CD4+ T cells	Flies et al. [12]

*AML* acute myeloid leukemia, *FFPE* formalin-fixed paraffin-embedded, *KO* knockout, *NSCLC* non-small cell lung cancer

### Melanoma

Melanoma is often used as a model to study the mechanism and the effect of immunotherapy in solid tumors. Blando et al. found a significantly increased density of memory T cells (CD45RO), B cells (CD20), cells expressing the activation markers ICOS and OX40, cytotoxic cells (Gr-B), and regulatory T cells (FoxP3) in melanoma tumors, and especially macrophage infiltration as defined by CD68 expression. The inhibitory checkpoint VISTA is predominantly expressed on macrophages, thus implicating VISTA as a potential immunotherapeutic target in melanoma [17]. Kakavand et al. also reported that the majority of melanoma patients showed a significantly increased proportion of VISTA+ lymphocytes following either treatment with anti-PD-1 alone or in with ipilimumab compared with the proportion detected prior to treatment [22]. Xu et al. used VISTA inhibitors to verify the function of VISTA as an inhibitory immune checkpoint in the B16-BL6 melanoma model [18]. Rosenbaum et al. observed that VISTA is expressed in melanoma patient samples and cell lines. Furthermore, tumor cell-specific expression of VISTA, which is regulated by factor forkhead box D3 (FOXD3), promotes tumor onset and enhances PD-L1 expression on tumor-infiltrating macrophages in vivo and is associated with increased intra-tumoral T regulatory cells [19]. There is some evidence that PD-L1/VISTA expression correlates with melanoma survival [19, 32, 33]. Recent trials have investigated the use of antibody combination therapy targeting VISTA. The effects of an antagonist anti-VISTA antibody appear to be non-overlapping with CTLA-4 and PD-1/PD-L1 pathways [20, 21], and some studies have shown that negative immune checkpoint regulation by VISTA represents an important potential mechanism of acquired resistance in melanoma patients treated with anti-PD-1 [22].

### Pancreatic cancer

Some studies on the expression of VISTA in pancreatic cancer tissue have demonstrated that VISTA is predominantly expressed and upregulated in the high-density-infiltrating immune cells but minimal in human pancreatic cancer (PC) cells, as well as the potential of VISTA as a critical target for pancreatic cancer immunotherapy [17, 23]. Recently, Blando et al. reported differential immune infiltration and inhibitory checkpoint expression in PC in comparison to melanoma and further demonstrated targeting VISTA as a promising immunotherapeutic strategy for patients with PC [17]. In brief, they found that (1) pancreatic tumors have a significantly higher density of VISTA, predominantly on CD68^+^ macrophages; (2) the engagement of the VISTA inhibitory pathway resulted in a greater decrease in CD8+ T cell responses than that achieved by the engagement of PD-L1 pathway; and (3) blockade of VISTA rather than PD-L1 inhibits cytokine production by tumor-infiltrating lymphocytes. Therefore, PD-L1 and VISTA represent separate inhibitory pathways that are capable of suppressing antitumor T cell responses in pancreatic cancer [17]. However, Byers et al. showed that VISTA staining was decreased or absent in pancreatic adenocarcinomas, and normal ducts adjacent to tumors were highly positive [24]. It was suggested that loss of the VISTA signal may contribute to immune evasion of pancreatic adenocarcinoma. Conversely, Liu et al. demonstrated that VISTA is minimally expressed in pancreatic cancerous cells but is not detected in either TME or normal pancreatic tissue. High-density infiltration of VISTA-upregulated immune cells was observed in PC [23]. Therefore, the immunoregulatory mechanism of VISTA in pancreatic adenocarcinoma requires further investigation.

### Prostate cancer

VISTA is a newly identified target for prostate cancer. Combination therapies including VISTA inhibitors have shown promising results in early-phase trials and it is likely that we will have an effective immunotherapy for advanced prostate cancer in the near future [34]. Gao et al. used ipilimumab to treat prostate cancer patients and found the level of VISTA inhibitory molecules had increased, especially on independent subsets of macrophages in tumors. They also investigated the expression of PD-L1 and VISTA on distinct subsets of CD68+ macrophages in post-treatment prostate tumor tissues. Based on these observations, it was concluded that the addition of anti-VISTA therapy to the currently available immune checkpoint inhibitors represents a new frontier in immunotherapy for prostate cancer although further studies are required to clarify the mechanism by which VISTA functions as an immunosuppressive checkpoint [14].

### Renal cell carcinoma

As for renal cell carcinoma (RCC), the clinical and pathological characteristics of the patients included in different studies have demonstrated that VISTA is predominantly expressed in CD45+ cells in para-tumor and tumor tissues. In other words, VISTA is expressed in hematopoietic tissues and highly expressed within the myeloid compartment [8, 26]. Based on studies showing that activated T cells are sensitive to VISTA-induced suppression, Ni et al. found that T cells obtained from kidney cancer patients were activated following binding of a VISTA-Fc fusion protein to surface Fc receptors [25]. While investigating PD-1-independent immune evasion mechanisms, Hong et al. discovered a high prevalence of VISTA expression in clear cell renal cell carcinoma (ccRCC) at both the mRNA and protein levels [26]. Their results also revealed that CD14+HLA-DR+ macrophages in the ccRCC tumors expressed higher levels of VISTA. Furthermore, the relationship of VISTA expression and CD8^+^ T cell responses identified in this study indicated that VISTA functions to suppress tumor immunity. Despite the limited number of studies on VISTA in RCC, existing evidence supports an inhibitory role for VISTA in its immune environment.

### Non-small cell lung cancer

There are many reports about the importance of VISTA in NSCLC. Villarroel-Espindola et al. investigated the relationship between VISTA protein levels and specific genomic alterations in lung adenocarcinomas by studying the differential distribution of VISTA expression in tumor and immune cells [13]. They also demonstrated that VISTA is frequently expressed in human NSCLC and shows an association with increased tumor-infiltrating lymphocytes, PD-1 axis markers, specific genomic alterations, and outcome. Hernandez-Martinez et al. verified the report by Espindola that VISTA plays an immunomodulatory role in human NSCLC, thus implicating its potential as a pivotal therapeutic target [27]. Brcic et al. found high numbers of regulatory T cells and VISTA expression on lymphocytes in samples of both squamous cell and adenocarcinomas of the lung [28]. In fact, cases with VISTA expression ≥ 10% had significantly higher numbers of Treg cells, indicating the potential influence of VISTA on immunosuppressive cells. These studies confirm the role of VISTA as an inhibitory immune checkpoint in NSCLC.

### Acute myeloid leukemia

In humans, VISTA is primarily found in hematopoietic tissues, with the highest expression in myeloid cells as well as lymphoid and myeloid dendritic cell populations [8]. In a study using a mouse model of acute myeloid leukemia (AML), the proliferation of leukemia cells was reduced in VISTA-knockout mice [29]. Leukemia growth was further diminished by treatment with a VISTA-blocking antibody in vivo. Wang et al. found that VISTA is highly expressed on myeloid-derived suppressor cells (MDSCs) in the peripheral blood, with a strong positive association between MDSC expression of VISTA and T cell expression of PD-1 in AML patients, despite an absence of evidence of direct regulation [30]. Evidence that VISTA has the highest expression in AML and induces immune evasion in acute myeloid leukemia has been presented at meetings [29, 35]. These observations suggest that VISTA expression by both AML and host cells can cause immune evasion, and support the strategy of VISTA-targeted treatment for AML while underscoring the strong potential for combined blockade of VISTA and PD-1 pathways in effective leukemia control.

### Colorectal cancer

There are many reports of high levels of VISTA expression in colorectal cancer, even exceeding the expression level of PD-1 in colorectal cancer [8, 15]. In addition, Zaravinos et al. revealed that CRC correlated with immune cytolytic activity (CYT) including immune checkpoints, and VISTA was expressed at significantly higher levels in microsatellite unstable colorectal cancers (MSI+ CRCs) compared to microsatellite-stable (MSS) tumors [36]. MSI+ CRCs expressing high VISTA levels responded strongly to anti-VISTA immunotherapy. Thus, these data imply the potential of VISTA as an inhibitory immune checkpoint in colorectal cancer immunotherapy. Under hypoxic conditions, hypoxia-inducible factors (HIFs) can be stabilized and promote tumor malignancy. Hypoxia promotes immune escape through deleterious metabolic and genetic adaptations in tumor cells. Tumor hypoxia is an independent negative prognostic factor that promotes resistance to therapy through multiple complex pathways [37, 38]. Xie et al. found that high VISTA expression is associated with worse overall survival of colorectal cancer patients and also identified a correlation between VISTA and HIF1α activity [15]. In other words, their data demonstrate a role for VISTA in immunosuppression that is specific to the TME and is likely to be driven by tumor hypoxia [15]. Other results indicate the clinical significance of VISTA in colorectal cancer [39]. Therefore, VISTA appears to promote immune system suppression in the tumor microenvironment.

### Ovarian and endometrial cancers

Mulati et al. reported that VISTA was expressed in 84 (91.3%) of 92 ovarian cancer tissues samples, with no difference in survival as a function of VISTA expression, probably due to the complex interaction between multiple immune checkpoint molecules and the weak suppressive function of VISTA in tumor cells. However, an anti-VISTA antibody prolonged the survival of tumor-bearing mice [31]. Liao et al. found that VISTA expression increased with advanced disease stage and lymph node metastasis (LNM), indicating that VISTA expression is involved in the progression of ovarian cancer [40]. Zong et al. concluded that VISTA expression in ovarian tumor cells was associated with a favorable prognosis in patients with high-grade serous ovarian cancer, and also closely related to the pathological type and PD-L1 expression [41]. In addition, VISTA mRNA expression was positively correlated with immune escape-modulating genes. In vitro studies by Mulati et al. showed that VISTA expression by tumor cells suppressed T cell proliferation and cytokine production resulting in immune evasion [31]. However, further investigations are required to elucidate the mechanism by which VISTA promotes tumor immune escape and verify its impact on survival in patients with ovarian and endometrial cancer. More importantly, these results implicate VISTA as a candidate immunotherapeutic target in ovarian and endometrial cancers.

### Glioma and fibrosarcoma

Although studies on immunosuppressive checkpoints in glioma and fibrosarcoma are scarce, the functions of VISTA as a negative immune checkpoint for T cell activation in glioma and fibrosarcoma tumor immunity have been described recently. Flies et al. discovered that VISTA-deficient animals were highly resistant to tumor induction in a murine brain glioma model [12]. Importantly, anti-CD4 mAb treatment-induced depletion of CD4+ T cells in vivo resulted in the elimination of tumor resistance in VISTA-KO mice treated with radiotherapy, whereas depletion of CD8+ T cells by the same mechanism had no impact on tumor growth or overall survival. Thus, it was concluded that VISTA selectively suppresses CD4+ T cell-mediated tumor immunity in this mouse glioma model. MCA105 (methylcholanthrene 105) fibrosarcoma does not express VISTA. Wang et al. proposed that VISTA overexpression on tumor cells interferes with protective antitumor immunity in the host based on the observation that VISTA-expressing MCA105 grew vigorously in vaccinated hosts, whereas the control tumors lacking VISTA expression failed to thrive [4].

Taken together, this evidence indicates that VISTA acts as an inhibitory immune checkpoint in multiple cancers, although the mechanism underlying its immunosuppressive function remains to be clarified in most cancer types except melanoma. Therefore, more detailed investigations are required to provide a better understanding of the comprehensive role of VISTA in the immunological inhibition of cancer.

## Co-stimulatory checkpoint-like roles of VISTA

The exact physiological mechanism of action of VISTA is still unclear. Some studies support the assumption that VISTA is an immune checkpoint receptor expressed on tumor-infiltrating T lymphocytes (TILs) and myeloid cells, leading to suppression of T cell activation, proliferation, and cytokine production and serves as an immune checkpoint [42, 43]. However, other studies have shown that VISTA is overexpressed in tumor tissues and functions as a co-stimulatory molecule [44–46]. Thus, we will discuss the stimulatory effects of VISTA on anti-cancer immunity in this part (Table 2).

**Table 2:** Stimulatory immune checkpoint-like effects of VISTA

Cancer type	Research object	VISTA expression	Reference
Ovarian cancer	Samples from patients with stage I–IV ovarian cancer	Tumor cells, immune cells, endothelial cells	Zong et al. [41]
Esophageal adenocarcinoma	Patient samples	Tumor cells; CD68+ TILs, CD4+ TILs	Loeser et al. [46]
Gastric cancer	Samples from patients with gastric cancer and corresponding liver metastases	Tumor cells, immune cells, endothelial cells	Boger et al. [45]
Hepatocellular carcinoma	Patient samples	Tumor cells, immune cells	Zhang et al. [47]

*TIL* tumor-infiltrating lymphocyte

### Esophageal adenocarcinoma and gastric cancer

In a recent study, Loeser et al. analyzed VISTA expression in a total of 393 esophageal adenocarcinomas (EAC) within a test-cohort and a validation-cohort using a monoclonal antibody (clone D1L2G) [46]. VISTA expression was detected on the tumor surface and infiltration margin, and VISTA-positive patients had a longer median overall survival compared to VISTA-negative patients. VISTA-positive tumors were found to be in the pT1/T2 stages, with a generally lower level of VISTA expression in pT3/T4 tumor samples. Tumors with VISTA-positive TILs demonstrated a significantly superior overall survival in early tumor stages (pT1/2) compared to patients without VISTA expression on TILs. However, the survival benefit was not seen in the more advanced tumor stages. In addition, Böger et al. showed that the number of VISTA-positive immune cells increased significantly from tumor category pT1 to pT2 and decreased significantly from pT2 to pT3 in gastric cancer (GCs) [45]. In addition, they found that VISTA-positive GCs had VISTA-negative liver metastases and vice versa. The mechanism responsible for the changes in VISTA expression in different tumor stages and metastatic disease remains to be clarified. It can be speculated that the biological activity of the tumor might reduce the amounts of VISTA-positive TILs in locally advanced tumors, or VISTA itself could influence invasive tumor growth [46]. Thus, both VISTA expression on TILs and tumor stage should be considered in the development of personalized immunotherapy based on the use of neutralizing antibodies against VISTA in humans.

### Hepatocellular carcinoma and ovarian cancer

In a study of VISTA protein expression in HCC, Zhang et al. [47] detected VISTA expression in 29.5% of HCC tissues, with 16.4% of tissues positive for tumor cells (TCs), and 16.9% positive for immune cells (ICs). VISTA expression was significantly associated with tissues with a high pathological grading (III–IV), without liver cirrhosis, and with a high density of CD8 + TILs. Patients with VISTA-positive staining in TCs, but not in ICs, showed significantly prolonged overall survival (OS) compared with those with VISTA-negative expression. Patients with VISTA-positive and CD8-positive staining showed a significantly longer OS than either VISTA-positive or CD8-positive patients, or both VISTA- and CD8-negative patients. VISTA expression was significantly correlated with the density of CD8 + TILs, indicating that VISTA affects signaling in the tumor microenvironment in a way that increases T cell infiltration. Furthermore, VISTA expression was found associated with prolonged OS in ovarian cancer patients. Zong et al. found that VISTA was expressed in TCs, ICs, and endothelial cells in ovarian cancer [41]. VISTA expression in ICs and all cells combined (TCs, ICs, and endothelial cells) was significantly more common in PD-L1-positive cells. VISTA expression in TCs alone was not associated with the expression level of PD-L1.

However, VISTA-positive staining in TCs, but not in ICs, was significantly associated with prolonged survival in patients with high-grade serous ovarian cancer. Studies by both Zhang et al. and Zong et al. showed that VISTA expression in TCs was associated with significantly prolonged OS, which implied that VISTA protein expression plays a role in inhibiting TC proliferation and progression. VISTA has been identified as a negative checkpoint regulator, and a potent suppressor of T cell proliferation and activation [8], which implies that VISTA expression is predictive of a poor prognosis.

In summary, in contrast to its inhibitory effects on the immune system, these studies suggest that high expression of VISTA is closely related to a favorable prognosis in several specific cancer types. Therefore, VISTA has the potential to function as a stimulatory checkpoint in anti-cancer immunity, and the mechanism is worthy of further investigation.

## Precise immunotherapeutic potential of VISTA in different cancers

To date, at least two clinical trials of VISTA-targeted cancer therapy are in progress (Table 3). JNJ-61610588 (CI-8993) is a human monoclonal antibody against VISTA with potential negative checkpoint regulatory and antineoplastic activities that are currently in a clinical trial in advanced cancer patients. This antibody inhibits VISTA signaling, abrogates the VISTA-induced suppression of T lymphocyte-mediated immune responses, enhances cytotoxic T cell responses against tumor cells, and inhibits tumor cell growth. This VISTA blockade approach has been used in clinical trial NCT02671955. It was anticipated that 150 patients would be enrolled in this study, with anticipated primary completion and study completion dates in April 2018. However, the number of actual enrollments was only 12, with an actual primary completion date in January 2017, and an actual study completion date in July 2017. No study results have yet been posted on ClinicalTrials.gov and there are no reports of the results of this clinical trial in the literature. In 2015, CA-170 was licensed as the first small drug-like molecule inhibitor that selectively targets PD-L1 and VISTA. Pre-clinical data revealed that CA-170 induced effective proliferation and IFN-γ production by T cells that are specifically suppressed by PD-L1 or VISTA. Meanwhile, a study of CA-170 is still currently being conducted in advanced solid tumors or lymphomas, although the trial coordinators are not recruiting and the last update was posted on May 6, 2019. The anticipated enrollment of this study is 300. However, the results of this CA-170 clinical trial have not yet been reported.

**Table 3:** Drug candidates targeting VISTA in clinical trials

Intervention	Condition(s)	Phase	Identifiers	Status	Location
JNJ-61610588 (CI-8993)	Advanced cancers	I	NCT02671955	Terminated	USA
CA-170	Advanced solid tumors or lymphomas	I	NCT02812875	Active, not recruiting	USA

To further clarify the therapeutic potential of the VISTA-targeting strategy in cancer immunotherapy, a series of genomic and immuno-omics analyses have been performed.

### Expression profile of VISTA

**Fig. 3:**
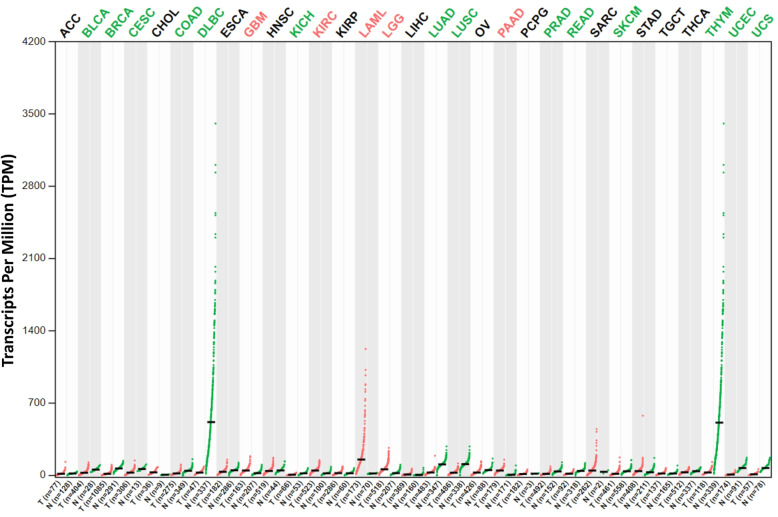
Expression profile analyses of VISTA across multiple cancers and normal tissues. Expression pattern of VISTA in ACC, BLCA, BRCA, CESC, CHOL, COAD, DLBC, ESCA, GBM, HNSC, KICH, KIRC, KIRP, LAML, LGG, LIHC, LUAD, LUSC, OV, PAAD, PCPG, PRAD, READ, SARC, SKCM, STAD, TGCT, THCA, THYM, UCEC, and UCS. GEPIA was used to generate dot plots profiling VISTA expression patterns across multiple cancer types (TCGA tumor) and paired normal tissue samples (TCGA normal + GTEx normal). Each dot represents the individual expression of a distinct tumor or normal sample. ANOVA method was used for differential gene expression analysis, and genes with higher |log2FC| values (> 1) and lower *q* values (< 0.01) were considered differentially expressed genes

According to the most recent reports and current knowledge as in this review, VISTA plays both positive and negative roles in tumor immunity. To better understand the potential roles and clinical relevance of VISTA in multiple human cancers, we first investigated the profiles of VISTA expression in 30 major human cancer types in The Cancer Genome Atlas (TCGA, http://cancergenome.nih.gov) database [48–50], using the Gene Expression Profiling Interactive Analysis (GEPIA, http://gepia2.cancer-pku.cn) package [51, 52]. In comparison to the healthy tissues, VISTA was expressed at obviously higher levels only in cholangiocarcinoma (CHOL), glioblastoma multiforme (GBM), kidney renal clear cell carcinoma (KIRC), acute myeloid leukemia (LAML), brain lower grade glioma (LGG), and pancreatic adenocarcinoma (PAAD) (Fig. 3). Intriguingly, significantly lower expression of VISTA was also observed in many other cancer types, including bladder urothelial carcinoma (BLCA), breast invasive carcinoma (BRCA), cervical squamous cell carcinoma and endocervical adenocarcinoma (CESC), colon adenocarcinoma (COAD), lymphoid neoplasm diffuse large B cell lymphoma (DLBC), kidney chromophobe (KICH), lung adenocarcinoma (LUAD), lung squamous cell carcinoma (LUSC), prostate adenocarcinoma (PRAD), rectum adenocarcinoma (READ), skin cutaneous melanoma (SKCM), uterine corpus endometrial carcinoma (UCEC), and uterine carcinosarcoma (UCS).

### Associations between VISTA, tumor-infiltrating lymphocytes, and immune-modulatory factors

**Fig. 4:**
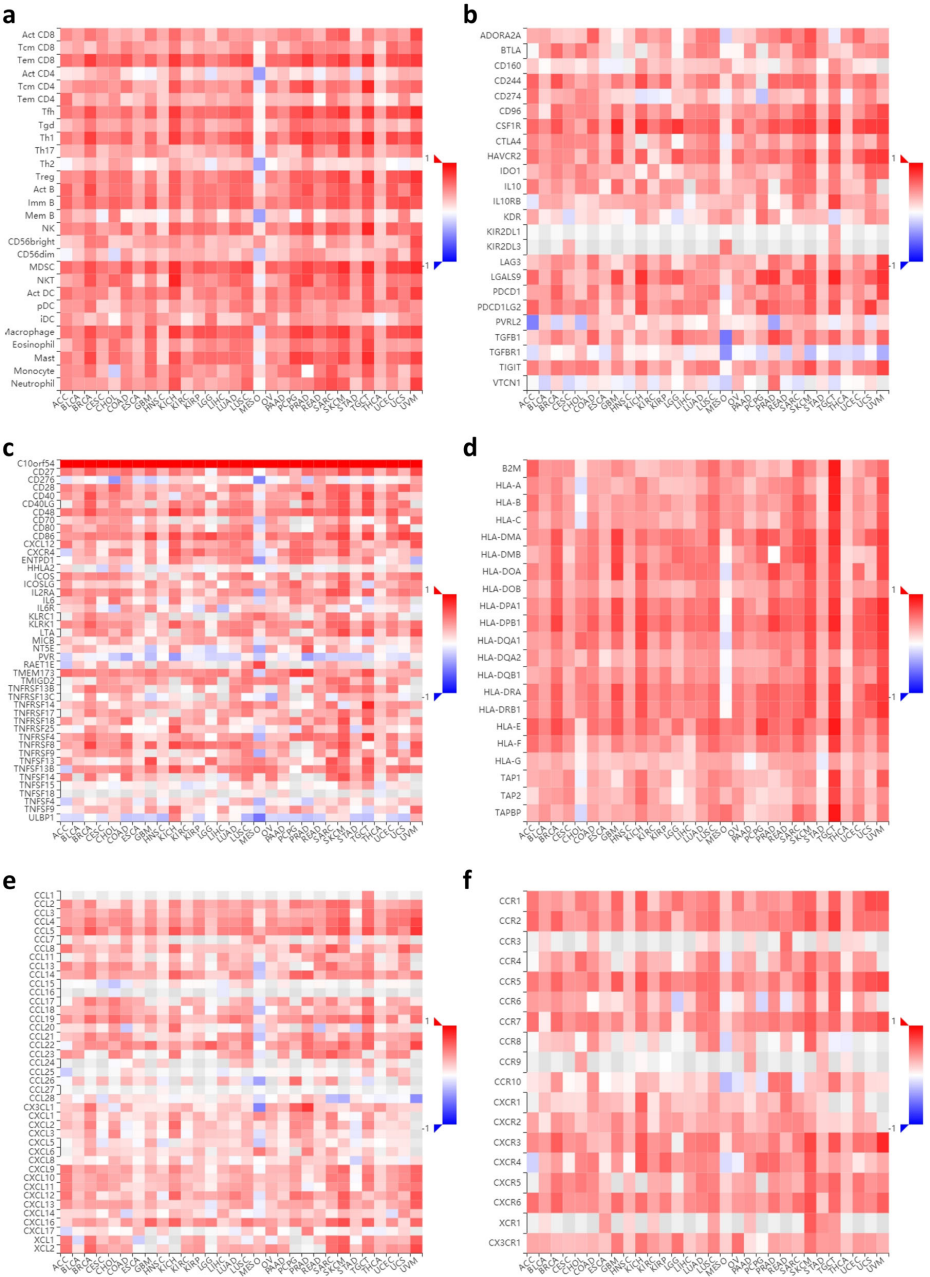
Correlation analyses between VISTA and immune regulation across multiple cancers. (**a**) Correlations between VISTA expression and the immune-related signatures of multiple tumor-infiltrating lymphocytes (TILs) across human cancers. (**b**) Correlations between VISTA expression and immunoinhibitors. (**c**) Correlations between VISTA expression and immunostimulators. (**d**) Correlations between VISTA expression and major histocompatibility complexes (MHCs). (**e**) Correlations between VISTA expression and chemokines. (**f**) Correlations between VISTA expression and chemokine receptors. TISIDB was used to generate correlations between expression of VISTA and abundance of TILs or immunomodulators across multiple cancers (TCGA tumor). For each cancer type, the relative abundances of TILs or immunomodulators were inferred by using gene set variation analysis based on gene expression profile. Each correlation between VISTA and a distinct TIL or immunomodulator in an individual cancer type was integrated into the indicated heatmap. Spearman method was used to analyze the pair-wise gene expression correlations, and *p* value < 0.05 was considered statistically significant

To further investigate the association between VISTA and cancer immunity, we utilized the Tumor and Immune System Interaction Database (TISIDB, http://cis.hku.hk/TISIDB) [44, 53] to analyze the potential relevance of VISTA in multiple immune regulatory cells and molecules across 30 cancer types. In accordance with previous reports about the contradictory roles of VISTA in cancer immunity, the outcomes of integrated immunological correlation analyses showed the following: (1) VISTA expression levels correlated positively with the relative abundance of almost all types of TILs with tumor-suppressing or tumor-promoting functions across 30 types of cancers, including but not limited to, activated CD8 T cells, natural killer cells, regulatory T cells, and MDSC (Fig. 4a). (2) VISTA expression levels correlated positively with the relative abundance of almost all critical immunomodulators regardless of their function as immunoinhibitors, immunostimulators, or major histocompatibility complexes (MHCs) across 30 types of cancers, including but not limited to, the critical immune checkpoints such as PD-1, PD-L1, CD80, and CD86 (Fig. 4b–d). (3) In addition, VISTA expression correlated positively with the relative abundance of almost all well-known chemokines and their receptors across 30 types of cancers, including but not limited to CXCL1, CXCL8, CXCL10, and CXCR3 (Fig. 4e, f).

### Associations between VISTA, prognosis, and clinical features

**Fig. 5:**
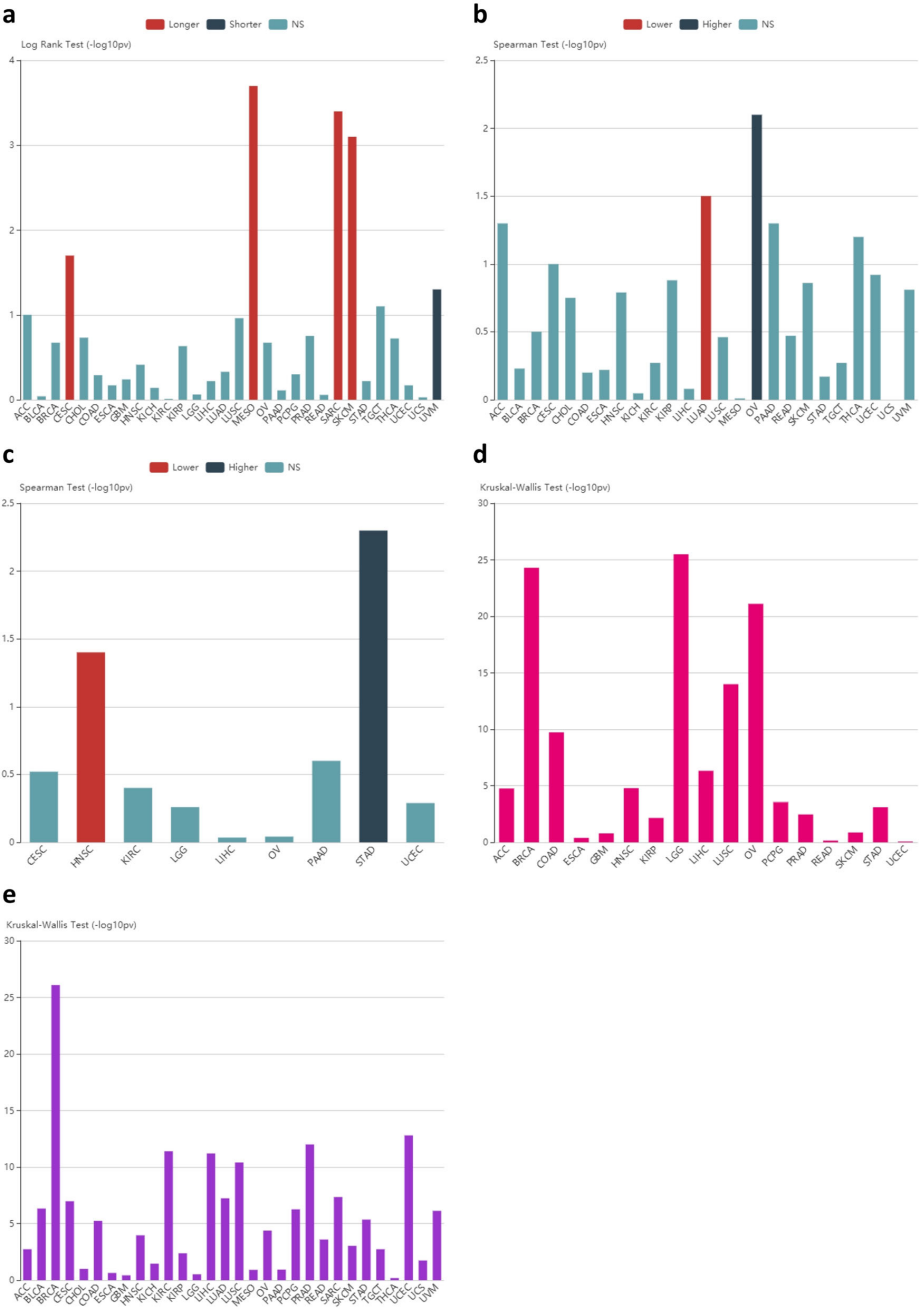
Association analyses between VISTA and clinical features across multiple cancers. (**a**) Associations between VISTA expression and overall survival across human cancers. (**b**) Associations between VISTA expression and stage across human cancers. (**c**) Associations between VISTA expression and grade across human cancers. (**d**) Associations between VISTA expression and molecular subtypes across human cancers. (**e**) Associations between VISTA expression and immune subtypes across human cancers. TISIDB was used to generate associations between expression of VISTA and prognostic impact or pathological distribution across multiple cancers (TCGA tumor). Log rank test and spearman test, as well as Kruskal-Wallis test, were individually used to calculate the associations, and *p* value < 0.05 was considered statistically significant

To fully clarify the clinical relevance of VISTA in terms of prognostic influence and pathological features, we further analyzed the association between VISTA and OS, TNM stage, and the tumor grades and subtypes across 30 cancer types using TISIDB. The results showed that (1) VISTA expression was associated with OS in only CESC, MESO, SARC, SKCM, and UVM across 30 cancer types, although the tendency was inconsistent (Fig. 5a). (2) VISTA expression was associated with tumor stages in only LUAD and OV across 30 cancer types, although the tendency was inconsistent (Fig. 5b). (3) VISTA expression was associated with the grades of only HNSC and STAD across 30 cancer types, although the tendency was inconsistent (Fig. 5c). (4) VISTA expression was associated with the molecular subtypes of only a few cancer types (Fig. 5d). (5) In addition, VISTA expression was associated with the immune subtypes of only some cancer types (Fig. 5e).

Overall, significantly higher or lower expression of VISTA has been observed in multiple human cancer types, and both correlated positively with immune effector cells and immune signatures, which further confirm the complex effects of VISTA on cancer immunity.

## Conclusions

In summary, after carefully considering the literature and further investigating the potential performance in the clinic, in this review, we summarized the up-to-date evidence plus the results of in silico analyses to highlight that VISTA acts as an inhibitory immune checkpoint in multiple cancer types, as well as its possible role as a stimulatory immune checkpoint. We further revealed the potential indeterminacy of the effects of application of anti-VISTA antibodies or VISTA-targeted strategies in clinical cancer treatment, which also warrant further discussion in relevant fields. According to the correlations between VISTA and prognosis or other parameters, VISTA functions in a tissue-specific manner, and thus we cannot conclude that VISTA functions as an inhibitory or stimulatory molecule. Therefore, based on the currently available information about VISTA, it is necessary to maintain a relatively conservative attitude regarding the prospect of targeting VISTA in cancer immunotherapy.

## Data Availability

Not applicable.

## Abbreviations

CTLA-4: Cytotoxic T lymphocyte-associated antigen-4
PD-1: Programmed death receptor
VISTA: V-domain immunoglobulin suppressor of T cell activation
NSCLC: Non-small cell lung cancer
FOXD3: Factor forkhead box D3
PC: Pancreatic cancer
PD-L1: Programmed death ligand 1
RCC: Renal cell carcinoma
ccRCC: Clear cell renal cell carcinoma
AML: Acute myeloid leukemia
MDSCs: Myeloid-derived suppressor cells
MSI+ CRCs: Microsatellite unstable colorectal cancers
HIFs: Hypoxia-inducible factors
LNM: Lymph node metastasis
MCA105: Methylcholanthrene 105
TILs: T lymphocytes
EAC: Esophageal adenocarcinomas
TCGA: The Cancer Genome Atlas
CHOL: Cholangiocarcinoma
GBM: Glioblastoma multiforme
KIRC: Kidney renal clear cell carcinoma
LGG: Brain lower grade glioma
PAAD: Pancreatic adenocarcinoma
BLCA: Bladder urothelial carcinoma
BRCA: Breast invasive carcinoma
CESC: Cervical squamous cell carcinoma and endocervical adenocarcinoma
COAD: Colon adenocarcinoma
DLBC: Lymphoid neoplasm diffuse large B cell lymphoma
KICH: Kidney chromophobe
LUAD: Lung adenocarcinoma
LUSC: Lung squamous cell carcinoma
PRAD: Prostate adenocarcinoma
READ: Rectal adenocarcinoma
SKCM: Skin cutaneous melanoma
UCEC: Uterine corpus endometrial carcinoma
UCS: Uterine carcinosarcoma
TISIDB: Tumor and Immune System Interaction Database
MHCs: Major histocompatibility complexes
